# Familial correlates of adolescent girls' physical activity, television use, dietary intake, weight, and body composition

**DOI:** 10.1186/1479-5868-8-25

**Published:** 2011-03-31

**Authors:** Katherine W Bauer, Dianne Neumark-Sztainer, Jayne A Fulkerson, Peter J Hannan, Mary Story

**Affiliations:** 1 Division of Epidemiology and Community Health, University of Minnesota, Minneapolis, MN, USA; 2 School of Nursing, University of Minnesota, Minneapolis, MN, USA

## Abstract

**Background:**

The family environment offers several opportunities through which to improve adolescents' weight and weight-related behaviors. This study aims to examine the cross-sectional relationships between multiple factors in the family environment and physical activity (PA), television use (TV), soft drink intake, fruit and vegetable (FV) intake, body mass index (BMI), and body composition among a sample of sociodemographically-diverse adolescent girls.

**Methods:**

Subjects included girls (mean age = 15.7), 71% of whom identified as a racial/ethnic minority, and one of their parents (dyad n = 253). Parents completed surveys assessing factors in the family environment including familial support for adolescents' PA, healthful dietary intake, and limiting TV use; parental modeling of behavior; and resources in the home such as availability of healthful food. Girls' PA and TV use were measured by 3-Day Physical Activity Recall (3DPAR) and dietary intake by survey measures. BMI was measured by study staff, and body fat by dual-energy X-ray absorptiometry (DXA). Hierarchical linear regression models tested individual and mutually-adjusted relationships between family environment factors and girls' outcomes.

**Results:**

In the individual models, positive associations were observed between family support for PA and girls' total PA (p = .011) and moderate-to-vigorous PA (p=.016), home food availability and girls' soft drink (p < .001) and FV (p < .001) intake, and family meal frequency and girls' FV intake (p = .023). Across the individual and mutually-adjusted models, parental modeling of PA, TV, and soft drink and FV intake was consistently associated with girls' behavior.

**Conclusions:**

Helping parents improve their physical activity and dietary intake, as well as reduce time watching television, may be an effective way to promote healthful behaviors and weight among adolescent girls.

## Background

One-third of adolescent girls in the United States are overweight or obese [1]. This high prevalence of overweight and obesity among adolescent girls, and specifically African American, Hispanic, and girls from low socioeconomic (SES) families [1,2], may be attributable to girls' participation in behaviors associated with higher weight and excess weight gain including lack of regular physical activity [3], frequent sedentary behavior including watching television [4], and poor dietary intake including frequent consumption of sugar-sweetened soft drinks [5] and less than adequate intake of fruits and vegetables [6].

Social Cognitive Theory postulates that mechanisms in individuals' social environments, including modeling of behavior, access or barriers to resources, reinforcement of behavior, and social norms for behaviors influence individuals' participation in behaviors that promote or harm health [7]. In support of this theory, researchers have begun to examine factors in the family environment that may play an important role in youths' weight and weight-related behaviors. For example, some studies have found associations between parental support and encouragement for physical activity [8] and healthy eating [9] and adolescents' physical activity and dietary intake. However, other studies have not found such associations [10]. Less is known about the relationship between parental encouragement for restricting television use and adolescents' television use [11]. Studies also suggest that parents' own television use [12] and dietary intake [13] are associated with adolescents' behavior, although studies examining relationships between parents' physical activity habits and adolescents' activity have produced inconsistent results [8,12,14]. Family meals have also emerged as an important factor in the family environment with adolescents whose families frequently eat meals together reporting better dietary intake [15]. Finally, studies suggest that physical resources in the home such as the number of televisions [16], having a television in youths' bedrooms [17], and having healthy or unhealthy food available in the home [18,19] contribute to adolescents' behavior.

Despite this growing body of literature, inconsistencies across studies and unaddressed questions limit our understanding of how to help families best support their daughters' physical activity, healthy dietary intake, and weight loss or maintenance. Specifically, many studies of the family environment have been conducted with predominantly white, higher SES samples, or among younger adolescents for whom the family may have a different influence on behavior as compared to older adolescents. Few studies have examined the family environment and its relationship with adolescents' weight-related behaviors among racially and ethnically diverse or lower SES adolescents within the United States, the youth who are most at risk for overweight and obesity. Also, few studies have assessed novel factors in adolescents' family environments such as the presence of physical activity equipment and media resources [20,21]. Finally, the majority of studies of the family environment utilize adolescents' report of their parents' behavior and home resources [10]. Adolescents' and parents' reports are often quite discrepant and parents' report of their own behaviors and the resources available in their home may be more valid than adolescents' report [22]. Understanding parents' perspectives is essential for the successful development and implementation of family-based obesity prevention interventions that aim to modify parental behavior and the home environment.

In order to fill these gaps in the literature, the objectives of the current study are to 1. Examine cross-sectional relationships between multiple factors in the parent-reported family environment and girls' physical activity (PA), television (TV) use, and soft drink and fruit and vegetable (FV) intake, and 2. Explore whether factors in the parent-reported family environment are associated with girls' body mass index (BMI) and body composition, and if so, whether these relationships are mediated by girls' physical activity, TV use, and soft drink and fruit and vegetable intake. This study utilizes a racially/ethnically and socioeconomically diverse sample of adolescent girls who are either currently overweight or obese, or at high risk for weight gain due to a sedentary lifestyle. Results can be used to inform the development of obesity prevention interventions that aim to improve adolescent girls' family environments, as well as provide guidance for conversations between clinicians and families about how to create a family environment that supports adolescents' healthy weight.

## Methods

### Study design

The current study is cross-sectional and data were drawn from the baseline assessment of adolescent girls (mean age = 15.7; range = 14.0 to 20.3) in grades 9 through 12 who participated in the New Moves intervention during either the 2007/2008 or 2008/2009 school year, and one parent of each girl. Of the 356 girls who participated in New Moves, 71% of their parents completed a survey assessing the family environment at baseline, resulting in 253 parent/girl dyads being included in the current study. New Moves was a school-based physical activity and nutrition intervention implemented primarily through an all-girls physical education class. Detailed information about the main intervention trial has been published elsewhere [23]. Twelve schools from 1 urban and 6 suburban school districts participated in the study. On average across the 12 schools 56% of students were eligible for free or reduced school breakfast and lunch. The New Moves intervention was advertised to all girls in the school and recruitment materials were designed to appeal to girls who were inactive and not comfortable being physically active, but who had a desire to be healthier. A short screening questionnaire developed for the current study was used to assess girls' frequency and duration of physical activity/exercise and their frequency of use of eating disorder behaviors (vomiting or laxative use weekly or more). Four girls were excluded because of high levels of physical activity (≥1 hour/day) and no girls were excluded because of eating disorder behaviors. Girls completed baseline data collection during either the end of spring semester or beginning of fall semester preceding their participation in New Moves. Data collection occurred either at the University of Minnesota's General Clinical Research Center or their school. The majority of parents completed surveys via mail, while 2% of parents completed the survey over the phone with the assistance of trained study staff. The study was approved by the University of Minnesota's Institutional Review Board and by each participating school district.

### Study sample description

The study sample was racially/ethnically diverse with 29% of girls reporting that they were white, 26% African American/black, 11% Hispanic, 24% Asian, 3% American Indian, and 8% of mixed race or another racial/ethnic group. Among the Asian girls, 87% percent identified as Hmong. Mothers comprised 79% of parent participants, 10% were fathers, and the remainder were other relatives or guardians. There was a diverse range of parental educational attainment with 28% of parents having not completed high school, 21% having only a high school diploma, 26% having attended some college, and 25% having completed college and/or post-graduate training. Approximately one-quarter of girls were born outside of the United States. Girls' BMIs ranged from 15 to 51. Approximately half of the girls (53%) were normal weight, while 17% were overweight and 30% were obese (Table 1).

**Table 1:** Sociodemographic characteristics of the New Moves Parent Project sample

	N	%
NMPP parent/daughter dyads	253	71.1^1^
**Parent Type**		
Mother	199	79.3
Stepmother	5	2.0
Other female guardian	6	2.4
Father	24	9.6
Stepfather	1	0.4
Other male guardian	3	1.2
Other guardian/relative	13	5.2
**Girls' Race/Ethnicity**		
White	74	29.3
African American/Black	65	25.7
Hispanic	27	10.7
Asian	61	24.1
American Indian	7	2.8
Mixed/Other	19	7.5
**Parents' Education Level**		
Did not finish high school	69	27.6
Finished HS/GED	52	20.8
Some college/training	66	26.4
College/University/Graduate Degree	63	25.2
**US-born Girls**	194	76.7
**Girls' weight-status**		
Normal weight	133	52.6
Overweight	44	17.4
Obese	76	30.0

^1 ^Percent of girls participating in New Moves (n = 356) who had a parent participate in the study

### Description of measures

#### Family environment measures

Selection of constructs to assess in the family environment was guided by Social Cognitive Theory [7], as well as previous research that identified components of the family environment associated with youths' weight and weight-related behaviors. Survey items were selected based on their psychometric qualities and were pilot tested by 10 parents of adolescents for applicability and comprehension.

##### Family Physical Activity (PA) Environment

Home availability of PA resources was assessed by parents' response to whether each of nine common types of exercise equipment (e.g. bicycle, exercise workout videotapes or DVDs, skis, or snowboards) was available in their home, yard, or apartment complex. This index has been found to have high construct validity [24]. Parental modeling of PA was determined by parents' response to three questions regarding the time spent participating in light, moderate, and vigorous PA per week. Both total PA and moderate-to-vigorous PA were examined. These items have shown acceptable validity when compared to individuals' VO_2 _max and body fat [25]. The items also had strong reliability with a 2-week test-retest r = 0.68 [25]. To assess family support for PA, parents responded to five questions regarding the frequency with which they or other members of their family provided various types of logistical and emotional support for physical activity to their daughter. Examples of types of support include providing transportation to a place where their daughter could participate in PA, participating in PA with their daughter, and telling their daughter she did well in a physical activity or sport. Response options ranged from "Never" to "Every day" on a 5-point Likert scale. The internal consistency of the parental support scale as measured by Cronbach's alpha was 0.78 and the 1-week test-retest was 0.81 [8].

##### Family Television (TV) use Environment

Media resources in the home were assessed by parents' response to whether they had each of five types of media resources (e.g. cable television and video/DVD player) in their home. The test-retest reliability of these categorical items has been reported to be high (percentage agreement = 91%-99%, K = 0.6-0.9) [26]. Parents were also asked to report how many televisions they had in their home. Response options ranged from 0 to 4 or more (ICC = 0.99) [26], and girls were asked whether they had a television in the room where they sleep [17]. Parents' television use was assessed with two questions regarding the number of hours they spend watching TV/videos/DVDs on weekdays and weekend days that were combined to produce the average weekly hours of parental TV use. Family support for limiting TV use was assessed with a single question regarding how often the parent encouraged their daughter to watch less TV. Response options ranged from "Never" to "Every day" on a 5-point Likert scale.

##### Family Food Environment

Parents reported on the availability of healthy food in the home with two questions regarding the frequency with which fruits and vegetables (FV) are available in the home, and two questions regarding how often FV are served at meals (Cronbach's α = 0.63, 2-week test-retest r = 0.54-0.59). Unhealthy home food availability was measured with three questions regarding the availability of soft drinks, salty snacks, and candy, and one question regarding the frequency with which soft drinks are served at meals (Cronbach's α = 0.80, 2-week test-retest r = 0.55-0.72) [18]. Parental modeling of fruit and vegetable intake was assessed with two questions regarding the number of servings of each type of food eaten on a typical day. Parents' soft drink intake was assessed with a single item regarding how many servings of regular soft drinks they drank in a typical week. Frequency of family support for healthy eating was assessed with a single item asking parents how often they or other family members encouraged their daughter to eat healthy food with response options ranging from "Never" to "Every day" on a 5-point Likert scale (2-week test-retest = 0.70) [9]. Weekly frequency of family meals (2-week test-retest = 0.74) and fast food for family meals were also each measured with a single item [27].

##### Physical activity and television use

Girls' total daily PA, daily moderate-to-vigorous physical activity (MVPA), and TV use were assessed using the 3-Day Physical Activity Recall (3DPAR). The 3DPAR has been shown to be a valid measure of MVPA as compared to accelerometry [28], and among adolescent girls had a 2-day test-retest reliability of r = 0.71 and r = 0.77 for MVPA and vigorous activity respectively [29]. The 3DPAR asks participants to recall the activities that they participated in during the majority of each 30-minute time block between 6 AM and midnight on the three days previous to the day of data collection. Girls selected the activity that they participated in for the majority of each half hour block from a list of 65 common sedentary behaviors and physical activities. Therefore, a block can be considered to be equivalent to between 15 and 30 minutes of activity as it is possible that the girls did not participate in the physical activity for the full half hour [29]. If a girl recorded engaging in a physical activity during a block, she was also asked to report whether her exertion level during that block was light, moderate, hard or very hard. For each physical activity at each exertion level, a corresponding metabolic equivalent (MET) value was identified [30]. Total PA was defined as a per day average of number of blocks for which any physical activity was reported. MVPA was defined as the per day average of number of blocks for which physical activities with a MET value greater than or equal to 3 were recorded [28]. TV use was determined by the average daily number of blocks during which subjects reported participating in "Watching TV or movies" [31].

##### Soft drink intake

Girls' intake of soft drinks was assessed with the following item: "Over the past month, how often did you drink regular soda pop (not diet)?" Response options included: "Never", "Less than once a week", "1-2 times per week", "3-4 times per week", "5-6 times per week", "1 time per day", "2 times per day", "3 times per day", "4 times per day", "5 or more times per day." These response options were adapted from an existing beverage intake item [32].

##### Fruit and vegetable intake

Girls' FV intake was assessed using the questions, "Thinking back over the past week, how many servings of fruit did you usually eat on a typical day? A serving would be a medium piece of fruit. Do not include juice." and "Thinking back over the past week, how many servings of vegetables did you usually eat on a typical day? A serving would 1/2 cup of cooked vegetables or 1 cup of raw vegetables. Do not include potatoes or French fries." Response options for both questions included: "None", "Less than 1 serving", "1 serving", "2 servings", "3 servings", "4 servings", and "5 or more servings" [18].

##### Body Mass Index (BMI)

Trained study staff measured each girl's body weight using a Tanita Body Composition Analyzer TBF-300A (Tanita Corporation of America, Arlington Heights, IL) and height using a portable stadiometer. BMI was calculated using the formula: weight in kilograms divided by height in meters squared. Girls' BMI percentiles were calculated using the 2000 CDC growth charts. Girls whose BMI was less than the 85th percentile were categorized as healthy weight, those whose BMI was between the 85th and 94th percentile were categorized as overweight, and those whose BMI was equal to or greater than the 95th percentile were categorized as obese.

##### Body composition

Girls' total percent body fat was assessed using a Lunar Prodigy dual-energy X-ray absorptiometry (DXA) apparatus (Lunar Radiation Corp., Madison, WI) at the University of Minnesota's General Clinical Research Center. The software for adults was used as the high school-aged girls participating in New Moves were all menstruating and close to full physical maturity. DXA has been found to be a highly valid and reliable measure of body fat [33].

### Statistical analysis

Hierarchical linear regression models were developed to examine the relationships between each of the family environment variables and girls' behaviors (PA, MVPA, TV, and soft drink and FV intake), and BMI and percent body fat represented as continuous variables. Family environment variables were standardized to have a mean of 0 and a standard deviation of 1 to allow for comparison of strength of regression coefficients across predictors for each outcome. Girls' age, race/ethnicity, and parental education were included as covariates in the models to reduce potential confounding. In order to account for potential clustering of behaviors among girls who attended school together, school was included in the regression models as a random effect [34]. The outcome variables of girls' total PA, MVPA, TV use, and soft drink intake were also square-root transformed to an approximate Gaussian distribution, and models examining relationships between the family environment factors and these transformed outcomes were developed. The strength and significance of resulting parameter estimates were similar in models with the transformed and non-transformed outcomes; therefore, models with the non-transformed outcomes were presented for ease of interpretation. In addition to examining the relationship between each family environment variable and the corresponding behavioral or body composition outcome, single regression models were developed to examine the total association between all of the outcome-specific family environment variables and the girls' outcomes. P < . 05 from a two-sided test of significance was used to direct attention to statistically significant results. In order to understand the explanatory power of the family environment on girls' outcomes, two models were developed to estimate the percent of variance (R^2^) in each outcome explained by (1) age, race/ethnicity, and parental education, and (2) age, race/ethnicity, parental education, and the family environment variables. The school-level variance was not partitioned from the R^2 ^making the estimate of variance accounted for by the model a conservative estimate. Analyses were conducted using SAS 9.2 (Cary, NC).

## Results

### Associations between the family environment and girls' behavioral outcomes

In independent analyses adjusted for race/ethnicity, parental education, and age, parents' total PA and family support for girls' PA were both significantly associated with girls' total PA (Table 2). Similar significant associations were seen between parents' MVPA and family support for girls' PA and girls' MVPA. The presence of PA resources including exercise equipment, bicycles, and workout DVDs was marginally associated with girls' PA and MVPA. In the mutually-adjusted model when parental modeling of MVPA, support for PA, and PA resources were included in the same model, parents' PA emerged as a statistically significant predictor of girls' MVPA. The sociodemographic characteristics of age, race/ethnicity, and parental education explained 2% of the variance in girls' total PA and the addition of the family environment variables to the model increased the explained variance to 8%. Similarly, sociodemographic characteristics explained 4% of girls' MVPA and the addition of the family environment variables to the model increased the explained variance to 8%.

**Table 2:** Associations between family environment factors and girls' total physical activity, moderate to vigorous physical activity, and television use

		**Independent Associations**^**a**^		**Mutually-adjusted Associations**^**b**^	
	n	Estimate	p	Estimate	p
**Outcome: Girls' total physical activity (PA) in 30-minute blocks/day**					
Home PA Resources	252	0.51	.067	0.29	.308
Parental Total PA	251	**0.59**	**.016**	0.48	.062
Family Support for PA	249	**0.63**	**.011**	0.41	.121
					
**Outcome: Girls' moderate-to-vigorous physical activity (MVPA) in 30-minute blocks/day**					
Home PA Resources	252	0.41	.063	0.22	.336
Parental MVPA	251	**0.48**	**.014**	**0.40**	**.047**
Family Support for PA	249	**0.47**	**.016**	0.28	.169
					
**Outcome: Girls' television (TV) use in 30-minute blocks/day**					
Media resources	251	0.19	.318	0.27	.182
Number of TVs in home	248	0.28	.125	0.04	.861
TV in bedroom	239	0.31	.136	0.25	.265
Parental TV use	248	**0.49**	**.008**	**0.43**	**.033**
Familial encouragement to decrease TV use	250	-0.14	.446	0.04	.836

Note: Estimates significant at the p < .05 level have been emboldened.

^a ^Models included sociodemographic variables (race/ethnicity, parental education, and age) and school as random effect.

^b ^Models included sociodemographic variables, school, and outcome-specific independent variables.

Parents' TV use was the only family environment variable significantly associated with girls' TV use in both the independent and mutually-adjusted models (Table 2). Media resources in the home, number of TVs in the home, the presence of a TV in girls' bedroom, and familial encouragement for their daughter to decrease TV were all unrelated to girls' TV use in both models. Sociodemographic characteristics explained 6% of the variance in girls' TV use and the addition of the family environment variables to this model increased the variance explained to 11%.

In the independent models, home availability of soft drinks and parents' soft drink intake were strongly positively associated with girls' soft drink intake (Table 3). These items remained independently predictive of girls' intake in the mutually-adjusted model. Sociodemographic characteristics explained 3% of the variance in girls' soft drink intake, and the addition of all of the family environment variables increased the variance explained to 15%. In the independent models, home availability of FV, parents' FV intake, familial encouragement to eat healthy food, and the frequency of family meals were all positively associated with girls' FV intake (Table 3). In the mutually-adjusted analysis, only parent's FV intake remained predictive of girls' intake. Sociodemographic characteristics explained 9% of girls' FV intake and the addition of the family environment variables increased the variance explained to 20%.

**Table 3:** Associations between family environment factors and girls' soft drink and fruit and vegetable intake

		**Independent Associations**^**a**^		**Mutually-adjusted Associations**^**b**^	
	n	Estimate	p	Estimate	p
**Outcome: Soft drink intake in servings/day**					
Home availability of FV	251	-0.11	.238	-0.09	.347
Home soft drink availability	251	**0.41**	**<.001**	**0.31**	**.003**
Parental soft drink intake	250	**0.44**	**<.001**	**0.30**	**.005**
Familial encouragement to eat healthy foods	249	0.03	.706	0.14	.156
Family meal frequency	252	0.05	.546	0.09	.308
Fast food family meal frequency	252	0.02	.782	-0.09	.310
					
**Outcome: Fruit and vegetable (FV) intake in servings/day**					
Home availability of FV	251	**0.71**	**<.001**	0.28	.174
Home availability of unhealthy food	247	0.06	.749	0.10	.574
Parental FV intake	251	**0.85**	**<.001**	**0.66**	**<.001**
Parental encouragement to eat healthy food	249	**0.53**	**.003**	0.28	.155
Family meal frequency	252	**0.41**	**.023**	0.08	.687
Fast food family meal frequency	252	0.10	.563	0.05	.773

Note: Estimates significant at the p < .05 level have been emboldened.

^a ^Models included sociodemographic variables (race/ethnicity, parental education, and age) and school as random effect.

^b ^Models included sociodemographic variables, school, and outcome-specific independent variables.

### Associations between the family environment and girls' BMI and percent body fat

The majority of behavior-specific family environment factors were not associated with girls' BMI or percent body fat in either the independent or mutually-adjusted models (Table 4). However, a positive relationship was observed between the number of media resources in the home and both girls' percent body fat and BMI. The positive association between number of media resources and percent body fat remained significant in the mutually-adjusted model after adjustment for other family environment factors. Additionally, girls with more televisions in their home were more likely to have a higher BMI, and family meal frequency was inversely associated with girls' BMI. Given that no significant associations were observed between girls' PA, TV, and dietary intake and girls' BMI or percent body fat (data not shown), and the lack of significant relationships between media resources and number of TVs in the home and girls' TV use, the observed relationships between the family environment factors and girls' BMI were not mediated by the behaviors assessed in the current study. Sociodemographic characteristics accounted for 6% of the variation in girls' BMI and 5% of the variation in girls' percent body fat. The inclusion of all of the family environment factors in the models raised the variance explained to 15% for BMI and 18% for percent body fat.

**Table 4:** Associations between family environment factors and girls' BMI and percent body fat^a^

	BMI units				Percent body fat			
	**Independent Associations**^**a**^		**Mutually-adjusted Associations**^**b**^		**Independent Associations**^**a**^		**Mutually-adjusted Associations**^**b**^	
	Estimate	p	Estimate	p	Estimate	p	Estimate	p
**PA-related Family Environment**								
Home PA Resources	0.24	.630	0.09	.892	0.33	.656	0.47	.618
Parental Total PA	0.03	.943	0.10	.924	0.17	.795	0.74	.597
Parental MVPA	0.08	.850	0.10	.918	0.11	.862	-0.29	.832
Family Support for PA	0.22	.623	0.09	.884	-0.45	.487	-1.28	.137
								
**Television-related Family Environment**								
Media resources	**1.04**	**.018**	0.68	.199	**2.19**	**.001**	**1.71**	**.024**
Number of TVs in home	**0.97**	**.030**	0.58	.322	1.01	.120	0.30	.719
Television in bedroom	0.74	.123	0.71	.215	0.21	.767	0.27	.746
Parental television use	0.67	.128	0.30	.576	0.52	.405	-0.07	.927
Familial encouragement to decrease television use	0.18	.689	-0.17	.759	0.30	.633	-0.32	.689
								
**Dietary intake-related Family Environment**								
Home availability of FV	-0.15	.728	0.19	.757	-0.28	.669	0.75	.393
Home availability of unhealthy food	-0.32	.472	-0.04	.964	-0.58	.368	0.27	.844
Home soft drink availability	-0.58	.211	-0.98	.347	-0.91	.180	-1.96	.198
Parental soft drink intake	0.26	.565	0.40	.510	0.57	.408	1.62	.077
Parental FV intake	-0.40	.376	-0.47	.386	-0.34	.599	0.41	.598
Familial encouragement to eat healthy food	0.52	.235	0.52	.394	0.24	.711	0.33	.713
Family meal frequency	**-0.99**	**.023**	-0.81	.117	-1.02	.112	-0.75	.311
Fast food family meal frequency	0.35	.410	0.33	.491	0.82	.181	0.48	.477

Note: Estimates significant at the p < .05 level have been emboldened.

^a ^Models included sociodemographic variables (race/ethnicity, parental education, and age) and school as random effect.

^b ^Models included sociodemographic variables, school, and independent variables.

## Discussion

The purpose of this study was to examine the cross-sectional associations between a wide range of characteristics of the family environment and girls' physical activity, television use and dietary behaviors, as well as to explore relationships between family environment factors and girls' BMI and body composition. Across all of the behavioral outcomes, in both the independent and mutually-adjusted analyses, parents' own behavior was associated with their daughters' behavior. The family environment factors observed in this study explained between 4 and 12 percent of the variance in girls' weight-related behaviors beyond the contribution of sociodemographic characteristics. Few associations were observed between the family environment factors and girls' BMI and body composition; however, the number of media resources in the home was positively associated with both girls' BMI and percent body fat.

Previous studies examining the relationship between parents' own PA habits and adolescents' activity have produced mixed results [8,35]. While in a longitudinal study utilizing parents' report of their physical activity habits, Anderssen et al [35] found that adolescents whose mothers were active exhibited less of a decline in physical activity through adolescence, most studies of adolescents have not found such a relationship. In their review of environmental correlates of PA in youth, Ferriera et al [10] noted that studies that utilized parental report of their own activity were more likely to show associations between parental PA and adolescents' activity, a factor that may have contributed to the associations between parental PA and girls' PA observed in the current study. Additionally, as girls who were not regularly physically active were specifically recruited for New Moves, it may be that sedentary girls are likely to have sedentary parents, while there may be a weaker association between parent and girl behavior among regularly-active girls.

The lack of association between family support for PA and girls' PA habits in the mutually-adjusted model was surprising considering a number of previous studies have found that family support and encouragement play an important role in the PA habits of adolescents [8,21,36]. However, most of these studies were conducted in primarily white and high SES samples [8,36]; therefore, familial support may be less influential to the PA habits of adolescents from lower SES and racial and ethnic minority groups. Additionally, few studies examined the combined association of parental modeling of behavior and parental support for behavior on girls' PA, even though often these two family characteristics are associated as parents who enjoy physical activity are likely to encourage their children to participate in activity. Despite previous suggestions that parental modeling may not be influential to adolescents' PA habits in the absence of parental support for PA [8], findings from the current study suggest that for socio-demographically diverse sedentary adolescent girls, parents' own physical activity habits may be more influential than the methods parents are using to encourage their daughter to be active. Reasons for this lack of an independent relationship may include that adolescent girls perceive parental encouragement as nagging, or are not receptive to parents' encouragement to be active when their parents themselves are not active. These findings support those of Heitzler et al [14] who observed that while simultaneously evaluating the relationships between family and peer factors and adolescents' PA, parents' PA was significantly associated with adolescents' PA while parental support for PA was not.

Despite previous studies observing multiple family environment predictors of youths' TV use [26,37], in the current study only parental TV use was associated with girls' TV use. This lack of consistency with other studies may be attributable to the fact that most previous studies utilized samples of grade school children or younger adolescents, whose behavior may be more influenced by parents' restriction of their TV time or by physical resources in the home. Additionally, for the current study girls who were sedentary, did not enjoy physical activity, and were at risk for obesity were actively recruited. These girls' TV use may be influenced by different social and environmental factors as compared to girls who are not sedentary. As the home is the venue at which most adolescents watch excessive TV, and modifying TV use has great potential to influence weight and body composition [38], identifying methods to intervene on adolescents' TV use is of vital importance.

Consistent with previous studies [13,15] factors in the family environment including parental intake of soft drinks and FV, home availability of these foods, and frequency of family meals were associated with girls' dietary intake in the independent models. However, only parental intake of soft drinks and FV and soft drink availability in the home remained independent predictors of girls' intake in the mutually-adjusted models. These findings suggest that the positive relationship observed between parents' and girls' intake is not merely due to the greater presence of these foods in the home because both parents and children eat them, but that dietary behavior can be instilled in youth through parental modeling of intake.

Few studies have examined relationships between behavior-specific family environment factors such as support for and modeling of physical activity and dietary intake, and adolescents' weight and body composition. In the current study, relationships were observed between media resources in the home and number of TVs in the home and girls' BMI, and between media resources in the home and girls' percent body fat in the independent models. This relationship was not mediated by girls' TV viewing. A significant inverse association was also found between the frequency of family meals and girls' BMI in the univariate models, a relationship that has been observed in previous cross-sectional studies [39]. This relationship was not mediated by girls' fruit and vegetable intake or by soft drink intake. This lack of mediation by television use and dietary intake suggests that family environment factors may influence weight and body composition via behaviors other than those assessed in this study. For example, the number of media resources and televisions in the home may be associated with girls' weight and body composition because adolescents spend time on the computer or playing video games at the expense of sleeping, an emerging risk factor for obesity [40].

This study addressed a number of gaps in the literature by examining the role of the family environment in girls' weight-related behaviors among a racially, ethnically, and socioeconomically diverse group of adolescent girls who were either currently overweight or obese or at risk for obesity due to a sedentary lifestyle. To confirm that this was a unique sample of sedentary girls, girls in the current study reported engaging in 3.0 blocks of MVPA per day and 7.9 blocks of sedentary activity (watching TV, listening to music, talking on the phone, using the computer, and hanging around) per day. This is less physical activity and slightly more sedentary activity than reported by a sample of African American and white 9^th ^grade girls who engaged in 3.5 blocks of MVPA and 7.8 blocks of sedentary activity per day [41]. While identifying a population of girls at high risk for overweight and obesity is a study strength, it is also a limitation in that study findings may not be highly generalizabile to other populations of adolescents. An additional strength of this study was the use of parental report of the family environment, which may be more valid than adolescents' report and highlights key areas for interventions aiming to modify parental behavior and the presence of resources in the home. Finally, use of both individual and mutually-adjusted models allowed for a comprehensive exploration of both the total effect of each of the family environment factors, as well as the unique contribution of each of the factors on youths' outcomes. Limitations of the current study include its cross-sectional design, which does not allow for an examination of the temporal relationship between family environment factors and girls' behavior, and the use of self-report measures for family environment factors as well as girls' behavior, which may be subject to reporting bias. Additionally, a limitation of the assessment of the family environment was while there was great breadth in constructs measured, in order to keep the survey at a reasonable length to ensure parent participation, in-depth assessment of some of the family components was sacrificed. Specifically, the lack of multi-item scales to assess some factors in the family environment, such as family support for healthy eating, is particularly limiting in studies such as this one that had a relatively small sample size. Having single-item measures coupled with a small study sample often results in large deviation around the mean, which may contribute to an inability to identify significant relationships when they do exist.

## Conclusions

Overall this study identified a number of future directions for research as well as potential intervention points to help parents create a family environment that supports adolescents' healthful eating and physical activity. Specifically, parents' behavior appears to play a significant role in youths' behavior. These results align with findings of obesity treatment intervention research by Epstein et al [42] and Golan et al [43] who through intervention trials found that modifying parents' PA and healthy eating habits resulted in greater weight improvements among children when compared to interventions in which only children, or parents and children together, were targeted for behavior change. Implementing programming to improve parental behavior would not only serve to increase the frequency of healthful behavior modeling in the home, but parents who are engaging in healthier behavior will likely make modifications to their home environment, such as increasing the availability of healthful food, that make it easier for children to make healthy choices. However, despite the number of significant associations observed between the family environment and girls' behavior and weight status, the family environment factors assessed in the current study accounted for only a small percentage of the variation in girls' behavior, suggesting that future research should examine the influence of both novel components of the family environment and factors outside of the home on girls' weight-related behaviors.

## Competing interests

The authors declare that they have no competing interests.

## Authors' contributions

KWB, DNS, JAF, PJH, and MS contributed to the study design, KWB contributed to data collection and conducted the statistical analysis and wrote the manuscript. All authors critically edited the manuscript and read and approved the final manuscript. DNS is the Principal Investigator of New Moves.
